# Crystal structure and photoreactive behaviour of *N*,*N*-diisoprop­yl(*p*-phenyl­phen­yl)glyoxyl­amide

**DOI:** 10.1107/S2056989021005387

**Published:** 2021-05-28

**Authors:** Hisakazu Miyamoto, Hiroki Takahashi

**Affiliations:** a Department of Liberal Arts (Sciences & Mathematics), National Institute of Technology, Kurume College, Fukuoka 830-8555, Japan; b Graduate School of Human and Environmental Studies, Kyoto University, Kyoto 606-8501, Japan

**Keywords:** crystal structure, photoreaction, chiral crystal

## Abstract

The title compound [systematic name: 2-([1,1′-biphen­yl]-4-yl)-2-oxo-*N*,*N*-bis(propan-2-yl)acetamide], C_20_H_23_NO_2_ was synthesized and its photoreactive properties in the crystalline state and in aceto­nitrile solution were investigated. The compound crystallizes in the chiral space group *P*2_1_2_1_2_1_. The crystal does not react under UV light irradiation, perhaps due to the presence of the biphenyl group. However, the compound is photoreactive in aceto­nitrile solution to give racemic 3-(*p*-phenyl­phen­yl)-3-hy­droxy-*N*-isopropyl-4,4-di­methyl­azetidin-2-one.

## Abstract 2

The photoreactive behaviour of the title compound, C_20_H_23_NO_2_, was investigated. Solid-state photoreaction did not occur in the solid-state but it occurred in aceto­nitrile solution.

## Chemical context   

**Figure 1:**
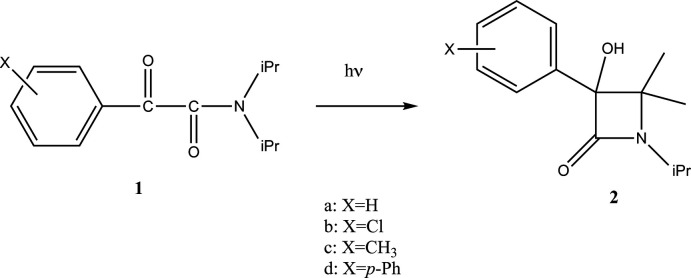
Photoreaction of *N*,*N*-diiso­propyl­aryl­glyoxyl­amide derivatives.

The solid-state photochemistry of *N,N*-dialkyl-α-oxo­amides has been studied in relation to penicillin chemistry (Aoyama *et al.*, 1979 ▸). The amides undergo Norrish type II cyclization giving β-lactams (Aoyama *et al.*, 1978 ▸). The achiral mol­ecule *N*,*N*-diiso­propyl­phenyl­glyoxyl­amide **1a** crystallizes in the chiral space group *P*2_1_2_1_2_1_ and is transformed to the optically active β-lactam derivative **2a** upon UV light irradiation (Fig. 1 ▸; Toda *et al.*, 1987 ▸; Sekine *et al.*, 1989 ▸). *N*,*N*-Diisoprop­yl(*m*-chloro or *m*-methyl or *o*-methyl­phen­yl)glyoxyl­amides **1b** and **1c** also form chiral crystals, and photoirradiation in the solid state gives optically active β-lactam derivatives **2b** and **2c**, respectively (Toda & Miyamoto, 1993 ▸; Hashizume *et al.*, 1995 ▸, 1996 ▸, 1998 ▸). However, *N*,*N*-diisoprop­yl(*p*-chloro or *o*-chloro or *p*-methyl­phen­yl)glyoxyl­amide **1b** and **1c** do not form chiral crystals, and their photoirradiation in the solid state gives racemic β-lactam derivatives **2b** and **2c**, respectively. Therefore, we synthesized the novel title compound **1d** having a phenyl group and investigated whether optically active β-lactam derivative **2d** could be obtained by photoreaction. It was found that **1d** formed a chiral crystal in the chiral space group *P*2_1_2_1_2_1_, but photoreaction did not proceed in the solid state. However, photoreaction of **1d** in aceto­nitrile solution proceeded to give racemic 3-(*p*-phenyl­phen­yl)-3-hy­droxy-*N*-isopropyl-4,4-di­methyl­azetidin-2-one **2d** in 26% yield. In this study, although **1d** formed a chiral crystal, the reason why the photoreaction product of **1d** in the solid state was not obtained was clarified by single-crystal X-ray structural analysis, UV spectroscopy and time-dependent density functional theory (TDDFT) calculations.
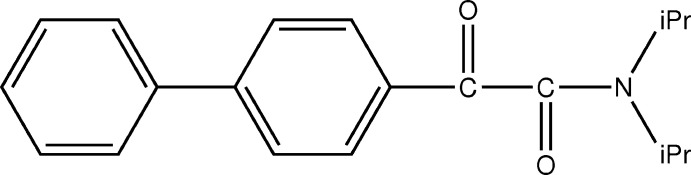


## Structural commentary   

**Figure 2:**
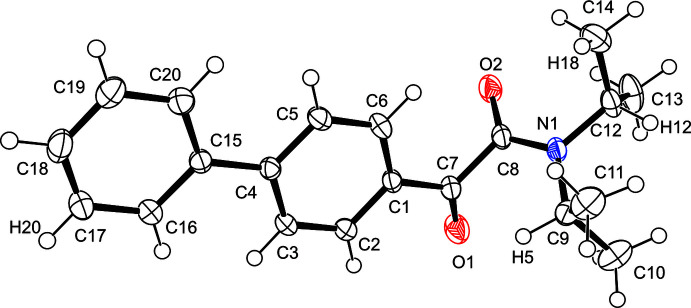
The mol­ecular structure of **1d**. Displacement ellipsoids for non-H atoms are drawn at the 50% probability level.

**Figure 3:**
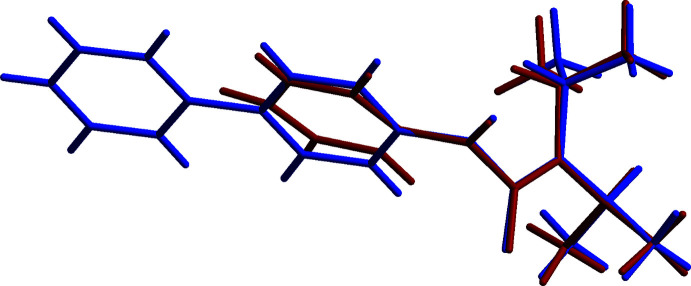
Overlay of mol­ecules **1a** (in red) and **1d** (in blue).

**Figure 4:**
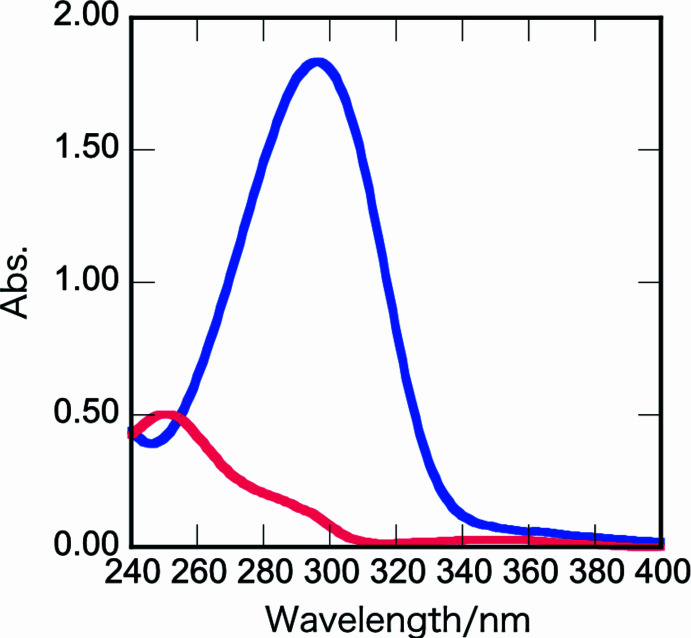
UV spectra of **1a** (in red, 51.3 *x* 10^−6^ *M* MeOH solution) and **1d** (in blue, 48.6 *x* 10^−6^ *M* MeOH solution).

Table 1 ▸ summarizes intra and inter­molecular hydrogen bonds observed in the title compound. The phenyl rings in the biphenyl group are coplanar with the carbonyl group (C7=O1). The torsion angles C2—C1— C7—O1 and C3—C4—C15—C16 are 7.8 (3) and −0.4 (2)°, respectively, and the torsion angles O1—C7—C8—O2 and C7—C8—N1—C9 are 97.1 (2) and −3.9 (2)°, respectively (Fig. 2 ▸). The corres­ponding torsion angles in **1a** are 88.0 (4) and −5.1 (4)°. In order for the Norrish–Yang reaction to take place, the reacting atoms in the mol­ecular structure must be in close proximity. The Yang cyclization of α-oxo­amides to β-lactams starts with abstraction of the γ-hydrogen (with respect to the benzylic carbon­yl) by the benzylic carbonyl oxygen in the excited state. In the title compound, there are two γ-hydrogen atoms (H5 on C9 and H12 on C12). The distances between the carbonyl oxygen atom O1 and the respective γ-hydrogen atoms H5 and H12 are 2.65 and 5.01 Å. The former inter­atomic distance is within the ideal value of up to about 2.7 Å, at which photoreaction can proceed in the crystal (Konieczny *et al.*, 2018 ▸). Moreover, the distance between the reacting C7 and C9 carbon atoms is 2.840 (2) Å, which is in the range of ideal values of up to about 3.2 Å. The corresponding distances are 2.78 (4) and 2.871 (4) Å in **1a**. As shown in Fig. 3 ▸, the geometries of the oxo­amide moiety of **1d** and **1a** are almost the same. Despite satisfying the geometry and distance requirements for the photoreaction, the corresponding β-lactam was not detected in the solid-state reaction. From the UV spectrum of **1d**, it is considered that the biphenyl group of **1d** absorbs ultraviolet light preventing the solid-state reaction (Fig. 4 ▸). In other words, the photocyclization reaction does not proceed in the solid state for at least 300 h because the irradiated UV light is absorbed by the π–π* transition of the biphenyl group.

## DFT calculations   

**Figure 5:**
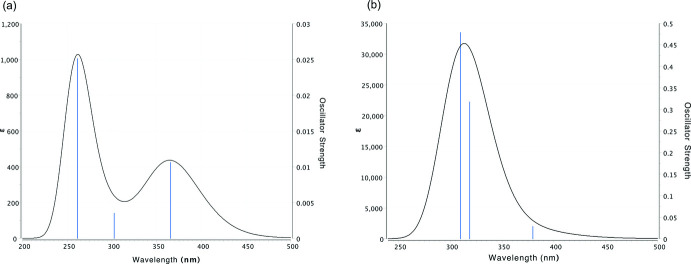
Calculated UV–vis spectra of (*a*) **1a** and (*b*) **1d**.

The *GAUSSIAN16* program (Frisch *et al.*, 2016 ▸) was used for density functional theory (DFT) calculations. Initial geom­etries of **1a** and **1d** were obtained from XRD data. Hydrogen atoms were optimized at the B3LYP/6–311G(d,p) level (Becke, 1993 ▸). The UV–vis spectra of **1a** and **1d** were calculated by the time-dependent density functional theory [TDDFT, B3LYP/6–311G(d,p)] method. In the calculated UV–vis spectra, there were two weak peaks at 254 and 362 nm for **1a**, and there was an intense and broad peak at 310 nm for **1d**. The calculated spectra were similar to the experimental spectra (Fig. 5 ▸). For **1a**, the peak at 254 nm corresponds to the π–π* transition of the Ph group, while that at the longer wavelength of 362 nm is due to *n*–π* transitions of the carbonyl groups. For **1d**, the adsorption peak at 376 nm was assigned to *n*–π* transitions of carbonyl groups. A very weak absorption peak was observed around 370 nm in the experimental spectrum. A mercury lamp has an intense emission at 365 nm, such that the photoreaction for **1a** proceeds rapidly in the solid state. In contrast, the large and broad absorption prevents the solid-state photoreaction for **1d**. Since the mol­ecules can move freely in solution, light irradiation for 60 h was uniformly performed, and it seemed that the reaction proceeded slightly. It has been reported that an oxo­amide derivative having a naphthyl group slows down the photoreaction (Natarajan *et al.*, 2005 ▸). The relationship between photoreactivity and irradiation wavelength is under investigation.

## Supra­molecular features   

**Figure 6:**
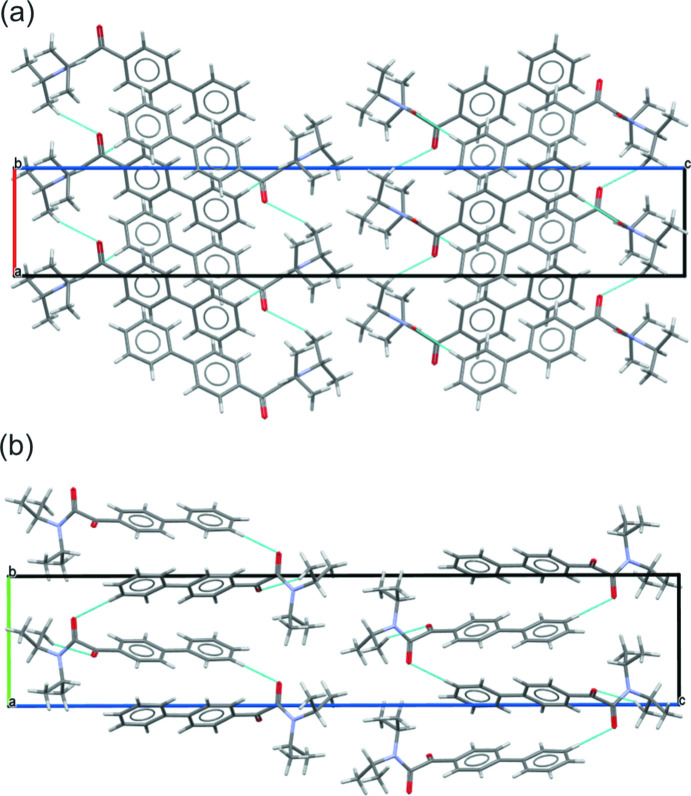
Packing diagrams for **1d** viewed (*a*) along the *b* axis and (*b*) along the *a* axis, showing inter­molecular C—H⋯O inter­actions as dotted blue lines.

In the crystal, the mol­ecules are linked by weak inter­molecular C—H⋯O (C14—H18⋯O1, 2.71 Å) inter­actions forming a 1D chain structure along the *a-*axis direction (Fig. 6 ▸ *a*), and C—H⋯O (C17—H20⋯O2, 2.66 Å) inter­actions forming a 1D zigzag chain structure along the *b-*axis direction (Fig. 6 ▸ *b*). Details of these inter­actions are given in Table 1 ▸.

## Database survey   

A search of the Cambridge Structural Database (Version 5.41, last update August 2020; Groom *et al.*, 2016 ▸) yielded 18 hits for compounds based on the *N*,*N*-diiso­propyl­phenyl­glyoxyl­amide fragment shown in Fig. 1 ▸: no substituent on the phenyl ring (JAGLAE; Sekine *et al.*, 1989 ▸), various chiral amido groups on the phenyl ring (KAHWIA, NAHZIG, NAHZUS, NAJBAC, NAJBEG, NAJBIK, NAJBOQ, NAJBUW, NAJCAD, and NAJCEH; Natarajan *et al.*, 2005 ▸), methyl or dimethyl group(s) on the phenyl ring (WIQKUC, YOWVUB, YOWVUF, and YOWWAI; Hashizume *et al.*, 1995 ▸), and a chlorine atom on the phenyl ring (ZOHNIT, ZOHNOZ, and ZOHNUF; Hashizume *et al.*, 1996 ▸).

## Synthesis and crystallization   

The title compound was prepared according to a reported method (Toda *et al.*, 1987 ▸; Sekine *et al.*, 1989 ▸), *i.e*., chlorination of 2-oxo-2-(4-phenyl­phen­yl)acetic acid with thionyl chloride followed by reaction with *N*,*N*-diiso­propyl­amine. Thus, to an ice-cooled solution of *N*,*N*-diiso­propyl­amine (16 mL, 0.11 mol) in dry diethyl ether (45 mL) was added a solution of 4-phenyl­benzoyl­formyl chloride (13.8 g, 0.0564 mol) in dry diethyl ether (45 mL), and the reaction mixture was stirred for 10 h at room temperature. After filtration of *N*,*N*-diiso­propyl­ammonium chloride, the filtrate was washed with dilute HCl and aqueous NaHCO_3_ and dried over MgSO_4_. The crude product was purified by silica gel column chromatography (toluene:ethyl acetate = 9:1) and recrystallized from toluene to give **1d** as colorless prisms (1.02 g, 5.8% yield, m.p. 397–398 K); IR (KBr): ν_max_ 1640, 1680 cm^−1^; ^1^H NMR (500 MHz, CDCl_3_): δ 8.01 (*d*, *J* = 8.0 Hz, 2H), 7.73 (*d*, *J* = 8.0 Hz, 2H), 7.63 (*d*, *J* = 8.0 Hz, 2H), 7.50–7.39 (*m*, 3H), 3.75 (*sept*, *J* = 6.9 Hz, 1H), 3.61 (*sept*, *J* = 6.9 Hz, 1H), 1.60 (*d*, *J* = 6.9 Hz, 6H), 1.20 (*d*, *J* = 6.6 Hz, 6H); ^13^C NMR (126 MHz, CDCl_3_): δ 190.7, 167.0, 147.1, 139.7, 132.1, 130.1, 129.0, 128.5, 127.6, 127.4, 50.2, 46.1, 20.6, 20.4; ESIMS *m*/*z*: calculated for C_20_H_23_NNaO_2_ [*M* + Na]^+^, 322.1621; found, 322.1586. Single crystals of **1d** suitable for X-ray diffraction analysis were grown from a toluene solution.

## Photoreaction   

**1d** (0.100 g, 0.323 mmol) was pulverized in a mortar and irradiated with a 400 W high-pressure mercury lamp for 300 h. No reaction took place, as determined by TLC, IR and NMR spectroscopies. **1d** (0.1368 g, 0.442 mmol) in aceto­nitrile (10 mL) was irradiated with a 400 W high-pressure mercury lamp for 60 h. The crude product was purified by silica gel column chromatography (toluene:ethyl acetate = 4:1) to give 3-(*p*-phenyl­phen­yl)-3-hy­droxy-*N*-isopropyl-4,4-di­methyl­aze­tidin-2-one **2d** as a colorless powder (0.035 g, 26% yield, m.p. 467-469 K); IR (KBr): ν_max_ 3200, 1720 cm^−1^; ^1^H NMR (60 MHz, CDCl_3_): δ 7.60–7.00 (*m*, 9H), 4.64 (*s*, 1H), 3.57 (*sept*, *J* = 7.0 Hz, 1H), 1.44 (*d*, *J* = 7.0 Hz, 6H), 1.27 (*s*, 3H), 0.87 (*s*, 3H); ESIMS *m*/*z*: calculated for C_20_H_23_NNaO_2_ [*M* + Na]^+^, 322.1621; found, 322.1569.

## Refinement   

Crystal data, data collection and structure refinement details are summarized in Table 2 ▸. All H atoms were positioned in geometrically calculated positions (C—H = 0.95–0.98 Å) and refined using a riding model with *U*_iso_(H) = 1.2*U*_eq_(C) and 1.5*U*eq(C-meth­yl). The Flack parameter *x* is 0.1 (4) as shown in Table 2 ▸. The standard uncertainty is large. The Flack and Hooft (Hooft *et al.*, 2008 ▸) parameters are strongly indicative of the correct absolute configuration, even when the standard uncertainties are large (Thompson & Watkin, 2011 ▸). Hooft [0.19 (16)] and Parsons parameters [0.2 (3)] (Parsons *et al.*, 2013 ▸) were calculated using *PLATON* (Spek, 2020 ▸).

## Supplementary Material

Crystal structure: contains datablock(s) I. DOI: 10.1107/S2056989021005387/dj2024sup1.cif

Structure factors: contains datablock(s) I. DOI: 10.1107/S2056989021005387/dj2024Isup2.hkl

Supporting information file. DOI: 10.1107/S2056989021005387/dj2024Isup3.cml

CCDC reference: 2085220

Additional supporting information:  crystallographic information; 3D view; checkCIF report

## Figures and Tables

**Table 1:** Hydrogen-bond geometry (Å, °)

*D*—H⋯*A*	*D*—H	H⋯*A*	*D*⋯*A*	*D*—H⋯*A*
C9—H5⋯O1	1.00	2.65	3.245 (2)	119
C13—H15⋯O2	0.98	2.47	3.033 (3)	117
C14—H16⋯O2	0.98	2.51	3.072 (3)	116
C14—H18⋯O1^i^	0.98	2.71	3.612 (3)	154
C17—H20⋯O2^ii^	0.95	2.66	3.608 (2)	179

Symmetry codes: (i) x-1, y, z; (ii) -x+1, y+{\script{1\over 2}}, -z+{\script{3\over 2}}.

**Table 2:** Experimental details

Crystal data	
Chemical formula	C_20_H_23_NO_2_
*M*_r_	309.39
Crystal system, space group	Orthorhombic, *P*2_1_2_1_2_1_
Temperature (K)	173
*a*, *b*, *c* (Å)	6.1313 (2), 7.3710 (2), 38.1143 (11)
*V* (Å^3^)	1722.53 (9)
*Z*	4
Radiation type	Mo *K*α
μ (mm^−1^)	0.08
Crystal size (mm)	0.31 × 0.29 × 0.29

Data collection	
Diffractometer	Rigaku Saturn 724+ CCD
Absorption correction	Numerical (*CrystalClear-SM Expert*; Rigaku, 2009 ▸)
*T*_min_, *T*_max_	0.985, 0.985
No. of measured, independent and observed [*I* > 2σ(*I*)] reflections	16094, 3877, 3614
*R*_int_	0.021
(sin θ/λ)_max_ (Å^−1^)	0.660

Refinement	
*R*[*F* ^2^ > 2σ(*F* ^2^)], *wR*(*F* ^2^), *S*	0.036, 0.080, 1.05
No. of reflections	3877
No. of parameters	212
H-atom treatment	H-atom parameters constrained
Δρ_max_, Δρ_min_ (e Å^−3^)	0.16, −0.15
Absolute structure	Flack *x* determined using 1346 quotients [(*I* ^+^)−(*I* ^−^)]/[(*I* ^+^)+(*I* ^−^)] (Parsons *et al.*, 2013 ▸)
Absolute structure parameter	0.1 (4)

Computer programs: *CrystalClear-SM Expert* (Rigaku, 2009 ▸), *SHELXT2014* (Sheldrick, 2015*a* ▸), *SHELXL2018* (Sheldrick, 2015*b* ▸), *Mercury* (Macrae *et al.*, 2020 ▸) and *publCIF* (Westrip, 2010 ▸).

## supplementary crystallographic information

### Crystal data

**Table:**

C_20_H_23_NO_2_	*D*_x_ = 1.193 Mg m^−^^3^
*M**_r_* = 309.39	Mo *K*α radiation, λ = 0.71073 Å
Orthorhombic, *P*2_1_2_1_2_1_	Cell parameters from 2939 reflections
*a* = 6.1313 (2) Å	θ = 3.4–27.5°
*b* = 7.3710 (2) Å	µ = 0.08 mm^−^^1^
*c* = 38.1143 (11) Å	*T* = 173 K
*V* = 1722.53 (9) Å^3^	Prism, colorless
*Z* = 4	0.31 × 0.29 × 0.29 mm
*F*(000) = 664	

### Data collection

**Table:**

Rigaku Saturn 724+ CCD diffractometer	3877 independent reflections
Radiation source: sealed tube	3614 reflections with *I* > 2σ(*I*)
Detector resolution: 28.5714 pixels mm^-1^	*R*_int_ = 0.021
profile data from ω–scans	θ_max_ = 28.0°, θ_min_ = 3.4°
Absorption correction: numerical (*CrystalClear-SM Expert*; Rigaku, 2009)	*h* = −8→7
*T*_min_ = 0.985, *T*_max_ = 0.985	*k* = −9→9
16094 measured reflections	*l* = −48→48

### Refinement

**Table:**

Refinement on *F*^2^	Hydrogen site location: inferred from neighbouring sites
Least-squares matrix: full	H-atom parameters constrained
*R*[*F*^2^ > 2σ(*F*^2^)] = 0.036	*w* = 1/[σ^2^(*F*_o_^2^) + (0.0314*P*)^2^ + 0.3778*P*] where *P* = (*F*_o_^2^ + 2*F*_c_^2^)/3
*wR*(*F*^2^) = 0.080	(Δ/σ)_max_ = 0.001
*S* = 1.05	Δρ_max_ = 0.16 e Å^−^^3^
3877 reflections	Δρ_min_ = −0.15 e Å^−^^3^
212 parameters	Absolute structure: Flack *x* determined using 1346 quotients [(*I*^+^)-(*I*^-^)]/[(*I*^+^)+(*I*^-^)] (Parsons *et al.*, 2013)
0 restraints	Absolute structure parameter: 0.1 (4)
Primary atom site location: difference Fourier map	

### Special details

**Table:**

Geometry. All esds (except the esd in the dihedral angle between two l.s. planes) are estimated using the full covariance matrix. The cell esds are taken into account individually in the estimation of esds in distances, angles and torsion angles; correlations between esds in cell parameters are only used when they are defined by crystal symmetry. An approximate (isotropic) treatment of cell esds is used for estimating esds involving l.s. planes.

### Fractional atomic coordinates and isotropic or equivalent isotropic displacement parameters (Å^2^)

**Table:**

	*x*	*y*	*z*	*U*_iso_*/*U*_eq_	
C1	0.5093 (3)	0.5728 (2)	0.66469 (4)	0.0240 (3)	
C2	0.6161 (3)	0.6491 (2)	0.69356 (4)	0.0264 (4)	
H1	0.757076	0.700514	0.690769	0.032*	
C3	0.5171 (3)	0.6499 (2)	0.72609 (4)	0.0256 (3)	
H2	0.591743	0.702783	0.745390	0.031*	
C4	0.3097 (3)	0.5750 (2)	0.73146 (4)	0.0231 (3)	
O1	0.8128 (2)	0.6133 (2)	0.62656 (3)	0.0413 (3)	
O2	0.4869 (2)	0.32629 (16)	0.59665 (3)	0.0381 (3)	
N1	0.4188 (2)	0.61084 (19)	0.57509 (4)	0.0276 (3)	
C5	0.2040 (3)	0.5007 (2)	0.70210 (5)	0.0282 (4)	
H3	0.062937	0.449217	0.704797	0.034*	
C6	0.3012 (3)	0.5011 (2)	0.66922 (4)	0.0287 (4)	
H4	0.225190	0.452194	0.649669	0.034*	
C7	0.6227 (3)	0.5697 (2)	0.63028 (4)	0.0284 (4)	
C8	0.4985 (3)	0.4927 (2)	0.59863 (4)	0.0281 (4)	
C9	0.4364 (3)	0.8099 (2)	0.58034 (5)	0.0318 (4)	
H5	0.497384	0.829750	0.604367	0.038*	
C10	0.5949 (4)	0.8947 (3)	0.55449 (6)	0.0470 (5)	
H6	0.736401	0.833410	0.556225	0.070*	
H7	0.612914	1.023717	0.559944	0.070*	
H8	0.537864	0.881506	0.530604	0.070*	
C11	0.2145 (4)	0.9013 (3)	0.57929 (6)	0.0424 (5)	
H9	0.156583	0.896594	0.555338	0.064*	
H10	0.229103	1.028114	0.586642	0.064*	
H11	0.114502	0.838140	0.595205	0.064*	
C12	0.3111 (3)	0.5445 (2)	0.54250 (4)	0.0309 (4)	
H12	0.270143	0.654227	0.528562	0.037*	
C13	0.4680 (4)	0.4345 (3)	0.51993 (5)	0.0466 (5)	
H13	0.601203	0.504937	0.515886	0.070*	
H14	0.398812	0.406781	0.497376	0.070*	
H15	0.504725	0.321214	0.532000	0.070*	
C14	0.1007 (4)	0.4442 (3)	0.55063 (6)	0.0434 (5)	
H16	0.134408	0.331101	0.563027	0.065*	
H17	0.024513	0.416204	0.528682	0.065*	
H18	0.007510	0.520425	0.565414	0.065*	
C15	0.2068 (3)	0.5729 (2)	0.76699 (4)	0.0242 (3)	
C16	0.3150 (3)	0.6450 (2)	0.79631 (5)	0.0327 (4)	
H19	0.454724	0.698538	0.793378	0.039*	
C17	0.2219 (4)	0.6397 (3)	0.82944 (5)	0.0385 (5)	
H20	0.299212	0.687471	0.848972	0.046*	
C18	0.0172 (4)	0.5653 (3)	0.83418 (5)	0.0387 (5)	
H21	−0.047408	0.563386	0.856828	0.046*	
C19	−0.0930 (3)	0.4937 (3)	0.80572 (5)	0.0360 (4)	
H22	−0.233437	0.441822	0.808872	0.043*	
C20	0.0005 (3)	0.4972 (2)	0.77263 (5)	0.0295 (4)	
H23	−0.077146	0.447082	0.753360	0.035*	

### Atomic displacement parameters (Å^2^)

**Table:**

	*U*^11^	*U*^22^	*U*^33^	*U*^12^	*U*^13^	*U*^23^
C1	0.0285 (8)	0.0234 (7)	0.0201 (8)	0.0024 (7)	−0.0035 (6)	0.0011 (6)
C2	0.0249 (8)	0.0275 (8)	0.0267 (9)	−0.0027 (7)	−0.0031 (7)	0.0002 (7)
C3	0.0275 (8)	0.0278 (8)	0.0216 (8)	−0.0033 (7)	−0.0061 (7)	−0.0022 (6)
C4	0.0267 (8)	0.0202 (7)	0.0223 (8)	0.0028 (7)	−0.0031 (6)	0.0009 (6)
O1	0.0359 (7)	0.0594 (9)	0.0286 (7)	−0.0063 (7)	0.0036 (6)	−0.0020 (6)
O2	0.0598 (9)	0.0259 (6)	0.0285 (6)	0.0030 (6)	−0.0079 (6)	0.0004 (5)
N1	0.0376 (8)	0.0255 (7)	0.0198 (7)	0.0002 (6)	−0.0016 (6)	−0.0001 (6)
C5	0.0252 (8)	0.0313 (8)	0.0283 (9)	−0.0048 (8)	−0.0037 (7)	−0.0017 (7)
C6	0.0303 (9)	0.0334 (9)	0.0224 (8)	−0.0006 (8)	−0.0081 (7)	−0.0034 (7)
C7	0.0338 (10)	0.0271 (8)	0.0244 (8)	0.0000 (7)	−0.0023 (7)	0.0021 (7)
C8	0.0365 (10)	0.0284 (8)	0.0194 (8)	0.0010 (8)	0.0007 (7)	0.0000 (7)
C9	0.0442 (11)	0.0264 (8)	0.0248 (9)	−0.0025 (8)	−0.0018 (8)	0.0011 (7)
C10	0.0446 (11)	0.0382 (11)	0.0581 (13)	−0.0029 (10)	0.0089 (10)	0.0104 (10)
C11	0.0506 (12)	0.0316 (9)	0.0451 (11)	0.0038 (9)	0.0135 (10)	−0.0045 (9)
C12	0.0421 (10)	0.0311 (9)	0.0195 (8)	0.0074 (8)	−0.0050 (7)	−0.0020 (7)
C13	0.0593 (14)	0.0570 (13)	0.0234 (9)	0.0184 (12)	−0.0023 (9)	−0.0081 (9)
C14	0.0476 (12)	0.0433 (11)	0.0395 (11)	−0.0028 (10)	−0.0093 (10)	−0.0081 (9)
C15	0.0293 (8)	0.0193 (7)	0.0240 (8)	0.0019 (7)	−0.0004 (7)	−0.0002 (6)
C16	0.0396 (10)	0.0323 (9)	0.0260 (9)	−0.0076 (8)	0.0005 (8)	−0.0016 (7)
C17	0.0568 (12)	0.0351 (10)	0.0238 (9)	−0.0080 (9)	0.0009 (9)	−0.0033 (8)
C18	0.0555 (13)	0.0322 (9)	0.0285 (9)	0.0005 (9)	0.0126 (9)	0.0031 (8)
C19	0.0360 (10)	0.0348 (9)	0.0373 (10)	−0.0009 (8)	0.0090 (9)	0.0038 (8)
C20	0.0305 (9)	0.0281 (8)	0.0300 (9)	0.0011 (8)	−0.0023 (7)	0.0000 (7)

### Geometric parameters (Å, º)

**Table:**

C1—C6	1.392 (3)	C11—H9	0.9800
C1—C2	1.398 (2)	C11—H10	0.9800
C1—C7	1.485 (2)	C11—H11	0.9800
C2—C3	1.380 (2)	C12—C14	1.519 (3)
C2—H1	0.9500	C12—C13	1.524 (3)
C3—C4	1.401 (2)	C12—H12	1.0000
C3—H2	0.9500	C13—H13	0.9800
C4—C5	1.404 (2)	C13—H14	0.9800
C4—C15	1.494 (2)	C13—H15	0.9800
O1—C7	1.217 (2)	C14—H16	0.9800
O2—C8	1.231 (2)	C14—H17	0.9800
N1—C8	1.342 (2)	C14—H18	0.9800
N1—C9	1.485 (2)	C15—C20	1.399 (3)
N1—C12	1.489 (2)	C15—C16	1.404 (2)
C5—C6	1.388 (2)	C16—C17	1.386 (3)
C5—H3	0.9500	C16—H19	0.9500
C6—H4	0.9500	C17—C18	1.382 (3)
C7—C8	1.535 (2)	C17—H20	0.9500
C9—C10	1.519 (3)	C18—C19	1.382 (3)
C9—C11	1.519 (3)	C18—H21	0.9500
C9—H5	1.0000	C19—C20	1.385 (3)
C10—H6	0.9800	C19—H22	0.9500
C10—H7	0.9800	C20—H23	0.9500
C10—H8	0.9800		
			
C6—C1—C2	118.98 (15)	H9—C11—H10	109.5
C6—C1—C7	122.22 (15)	C9—C11—H11	109.5
C2—C1—C7	118.80 (15)	H9—C11—H11	109.5
C3—C2—C1	120.18 (15)	H10—C11—H11	109.5
C3—C2—H1	119.9	N1—C12—C14	111.50 (15)
C1—C2—H1	119.9	N1—C12—C13	111.46 (16)
C2—C3—C4	121.88 (15)	C14—C12—C13	113.10 (17)
C2—C3—H2	119.1	N1—C12—H12	106.8
C4—C3—H2	119.1	C14—C12—H12	106.8
C3—C4—C5	117.15 (15)	C13—C12—H12	106.8
C3—C4—C15	121.29 (14)	C12—C13—H13	109.5
C5—C4—C15	121.56 (15)	C12—C13—H14	109.5
C8—N1—C9	121.65 (14)	H13—C13—H14	109.5
C8—N1—C12	120.38 (14)	C12—C13—H15	109.5
C9—N1—C12	117.97 (14)	H13—C13—H15	109.5
C6—C5—C4	121.36 (16)	H14—C13—H15	109.5
C6—C5—H3	119.3	C12—C14—H16	109.5
C4—C5—H3	119.3	C12—C14—H17	109.5
C5—C6—C1	120.44 (15)	H16—C14—H17	109.5
C5—C6—H4	119.8	C12—C14—H18	109.5
C1—C6—H4	119.8	H16—C14—H18	109.5
O1—C7—C1	123.19 (16)	H17—C14—H18	109.5
O1—C7—C8	118.74 (16)	C20—C15—C16	117.12 (16)
C1—C7—C8	117.87 (15)	C20—C15—C4	121.66 (15)
O2—C8—N1	125.77 (17)	C16—C15—C4	121.21 (16)
O2—C8—C7	116.41 (16)	C17—C16—C15	121.34 (18)
N1—C8—C7	117.78 (15)	C17—C16—H19	119.3
N1—C9—C10	111.42 (16)	C15—C16—H19	119.3
N1—C9—C11	111.71 (16)	C18—C17—C16	120.26 (18)
C10—C9—C11	111.97 (16)	C18—C17—H20	119.9
N1—C9—H5	107.1	C16—C17—H20	119.9
C10—C9—H5	107.1	C17—C18—C19	119.54 (17)
C11—C9—H5	107.1	C17—C18—H21	120.2
C9—C10—H6	109.5	C19—C18—H21	120.2
C9—C10—H7	109.5	C18—C19—C20	120.33 (19)
H6—C10—H7	109.5	C18—C19—H22	119.8
C9—C10—H8	109.5	C20—C19—H22	119.8
H6—C10—H8	109.5	C19—C20—C15	121.40 (17)
H7—C10—H8	109.5	C19—C20—H23	119.3
C9—C11—H9	109.5	C15—C20—H23	119.3
C9—C11—H10	109.5		
			
C6—C1—C2—C3	1.2 (2)	C1—C7—C8—N1	104.20 (19)
C7—C1—C2—C3	−178.37 (15)	C8—N1—C9—C10	110.4 (2)
C1—C2—C3—C4	0.3 (2)	C12—N1—C9—C10	−69.1 (2)
C2—C3—C4—C5	−1.0 (2)	C8—N1—C9—C11	−123.57 (18)
C2—C3—C4—C15	178.55 (15)	C12—N1—C9—C11	57.0 (2)
C3—C4—C5—C6	0.2 (3)	C8—N1—C12—C14	65.9 (2)
C15—C4—C5—C6	−179.27 (16)	C9—N1—C12—C14	−114.69 (18)
C4—C5—C6—C1	1.2 (3)	C8—N1—C12—C13	−61.6 (2)
C2—C1—C6—C5	−1.9 (2)	C9—N1—C12—C13	117.88 (18)
C7—C1—C6—C5	177.64 (16)	C3—C4—C15—C20	−179.53 (16)
C6—C1—C7—O1	−171.73 (18)	C5—C4—C15—C20	−0.1 (2)
C2—C1—C7—O1	7.8 (3)	C3—C4—C15—C16	−0.4 (2)
C6—C1—C7—C8	3.1 (2)	C5—C4—C15—C16	179.07 (17)
C2—C1—C7—C8	−177.40 (15)	C20—C15—C16—C17	0.6 (3)
C9—N1—C8—O2	178.42 (19)	C4—C15—C16—C17	−178.57 (17)
C12—N1—C8—O2	−2.2 (3)	C15—C16—C17—C18	−1.1 (3)
C9—N1—C8—C7	−3.9 (2)	C16—C17—C18—C19	1.0 (3)
C12—N1—C8—C7	175.50 (16)	C17—C18—C19—C20	−0.3 (3)
O1—C7—C8—O2	97.1 (2)	C18—C19—C20—C15	−0.2 (3)
C1—C7—C8—O2	−77.9 (2)	C16—C15—C20—C19	0.0 (2)
O1—C7—C8—N1	−80.7 (2)	C4—C15—C20—C19	179.20 (16)

### Hydrogen-bond geometry (Å, º)

**Table:**

*D*—H···*A*	*D*—H	H···*A*	*D*···*A*	*D*—H···*A*
C9—H5···O1	1.00	2.65	3.245 (2)	119
C13—H15···O2	0.98	2.47	3.033 (3)	117
C14—H16···O2	0.98	2.51	3.072 (3)	116
C14—H18···O1^i^	0.98	2.71	3.612 (3)	154
C17—H20···O2^ii^	0.95	2.66	3.608 (2)	179

Symmetry codes: (i) *x*−1, *y*, *z*; (ii) −*x*+1, *y*+1/2, −*z*+3/2.
